# Psychological and emotional benefits of virtual reality-based exercise for postpartum women with lumbopelvic pain: A randomized controlled trial

**DOI:** 10.1371/journal.pgph.0006354

**Published:** 2026-09-02

**Authors:** Javeria Aslam, Alba Paris-Alemany, Carlos Romero-Morales, Raquel Díaz- Meco Conde

**Affiliations:** 1 Faculty of Medicine, Health and Sports, Universidad Europea de Madrid, Villaviciosa de Odón, Madrid, Spain; 2 Department of Doctor of Physical Therapy, Shalamar School of Allied Health Sciences, Shalamar Institute of Health Sciences, Lahore, Pakistan; 3 Department of Basic Health Sciences, Health Science Faculty, Universidad Rey Juan Carlos, Alcorcon, Madrid, Spain; 4 Motion in Brains Research Group, CSEU La Salle, Universidad Autónoma de Madrid, Madrid, Spain; 5 Instituto de Dolor Craneofacial y Neuromusculoesquelético (INDCRAN), Madrid, Spain; University of Balamand Faculty of Health Sciences, LEBANON

## Abstract

Women experiencing the postpartum stage often develop psychological distress, fear of movement, and maladaptive pain coping, which is a hindrance to healing lumbopelvic pain (LPP). Traditional exercise (TE) programs are usually characterized by low adherence. Virtual reality (VR) - based exercise can be a strong immersive experience, which can positively affect emotional well-being, decrease fear of pain, and increase coping mechanisms. To determine the level of effectiveness of VR-based exercise programs as compared to TE in enhancing psychological and emotional outcomes in postpartum women with LPP. 60 postpartum women aged 20–40 years with LPP, registered between 2–12 weeks post-delivery, were enrolled in a single blind randomized controlled trial. The project followed a simple randomization process and was conducted computationally with an equal distribution (1:1) between the VR and TE groups. Both of the groups were exposed to 8 sessions of pelvic floor muscle training over a month. The VR group additionally performed immersive tasks using Meta Quest 2 headsets and Lumbar Pain Rehab software. Outcomes were measured at baseline, 4^th^ week, and 8^th^ week using the Depression, Anxiety, and Stress Scale (DASS), Pain Catastrophizing Scale (PCS), Coping Strategies Questionnaire (CSQ), Tampa Scale for Kinesiophobia (TSK), and Simulator Sickness Questionnaire (SSQ). The ethical approval was acquired (IRB no.0543; reference no. SMDC-IRB/AI/18–1/2023). The VR group demonstrated significantly better reductions in depression, anxiety, and stress at the 4^th^ week, alongside improved adaptive coping strategies and decreased pain catastrophizing compared with TE. These advantages were sustained at the 8^th^ week, with further improvement in kinesiophobia and a continued enhancement of emotional well-being. No adverse VR-related effects were reported. VR-based exercise demonstrated superior benefits over TE in enhancing psychological well-being, reducing fear of movement, and promoting adaptive coping strategies in postpartum women with LPP. ClinicalTrials.gov Identifier: NCT05921747, registered on 4/17/2023.

## Introduction

The postpartum period is characterized by highly complex physiological, psychological, and social readjustments, and this may predispose women to emotional instability and psychological suffering [1]. The effects of such modifications can be seen on general well-being and functional recovery by regulating the effects of pain, physical performance, and rehabilitation adherence. Specifically, it may be impaired by emotion-based reactions and fear-avoidant behaviour, which are reported to be predictors of chronic postpartum lumbopelvic pain (LPP) [2]. This state of vulnerability is created by a complex interaction of hormonal changes, physical stressors like sleep deprivation, pain, and psychosocial stress of nursing an infant.

Anxiety, depressive symptoms, emotional pain, and maladaptive patterns of thought are the most frequent psychological symptoms [3]. The discomfort in women who experience postpartum LPP is not only sensory. Nonetheless, it is highly influenced by emotional reactions, coping behaviour, as well as mental processes, which might affect recuperation and participation in routine endeavours [4]. These elements, commonly studied in pain-related rehabilitation studies, are not new in increasing the duration of disability with avoidance behaviors, pain sensitivity, and poor attendance of rehabilitation programs [5].

The traditional methods of treating LPP during the postpartum period focus on physical impairment and rely on pelvic floor exercises, core exercises, and strengthening exercises. Even though these interventions will prove to help return functionality and reduce the pain, it is not likely to directly incorporate the psychological and emotional elements of recovery. In order to improve general well-being, educational, cognitive-behavioral, relaxation, mindfulness, and supportive counselling interventions can be integrated with exercise programs [6]. These strategies can be used together to increase the outcome of coping, emotional control, and rehabilitation, particularly among women with postpartum symptoms of depression [7].

Virtual reality (VR)-based exercise has developed as an encouraging rehabilitation modality that combines physical exercise with immersive and interactive settings [8]. In addition to an enhanced physical condition, VR can have a positive impact on a psychological state, offering attentional distraction, movement grading, and cognitive involvement [9]. Although most evidence comes from other clinical populations, VR interventions have demonstrated efficacy in reducing pain and anxiety, and may therefore offer meaningful psychological and emotional benefits for postpartum women [10].

Hence, the purpose of this study was to compare the relative effectiveness of a VR-based exercise program with a traditional exercise (TE) towards addressing psychological and emotional factors associated with postpartum LPP. Outcomes were evaluated across scales of movement-related fear, maladaptive cognitive responses, emotional distress, coping, and simulator-related side effects. Given the immersive and engaging nature of VR, we proposed that physical activities in VR would produce overall better psychological and emotional responses.

## Materials and methods

### Ethics statement

Ethical approval was obtained from the Institutional Review Board of Shalamar Medical and Dental College (IRB no. 0543; reference no. SMDC-IRB/AI/18–1/2023). The study was conducted in accordance with the Declaration of Helsinki. Written informed consent was obtained from all participants prior to enrolment.

Consent documents and participant information sheets were provided in Urdu (local language) and explained verbally by trained researchers to ensure full understanding before participation.

Participant recruitment, screening, and data collection were conducted at Shalamar Hospital, Lahore, under supervision of the research team. Study design and analysis involved collaboration between local (Pakistan-based) and international investigators.

No formal community advisory board or patient/public involvement group was involved in the design of the study; however, the research question was developed based on clinical needs observed in routine postpartum rehabilitation practice.

The trial was registered at ClinicalTrials.gov (NCT05921747).

Additional information regarding the ethical, cultural, and scientific considerations specific to inclusivity in global research is included in the Supporting Information (S1 Checklist).

### Study design

This single-blind randomized controlled trial involved 60 postpartum women aged 20–40 years old diagnosed with LPP. Eligible participants who met the inclusion criteria were recruited.

**Randomization:** Group allocation followed a simple randomization procedure generated computationally, ensuring equal distribution (1:1) between the VR and TE groups. Allocation concealment was maintained using 60 sequentially numbered, opaque, and sealed envelopes prepared by an independent researcher. After enrollment, each envelope was opened in sequence to assign participants, ensuring balanced group sizes.

**Fig 1:**
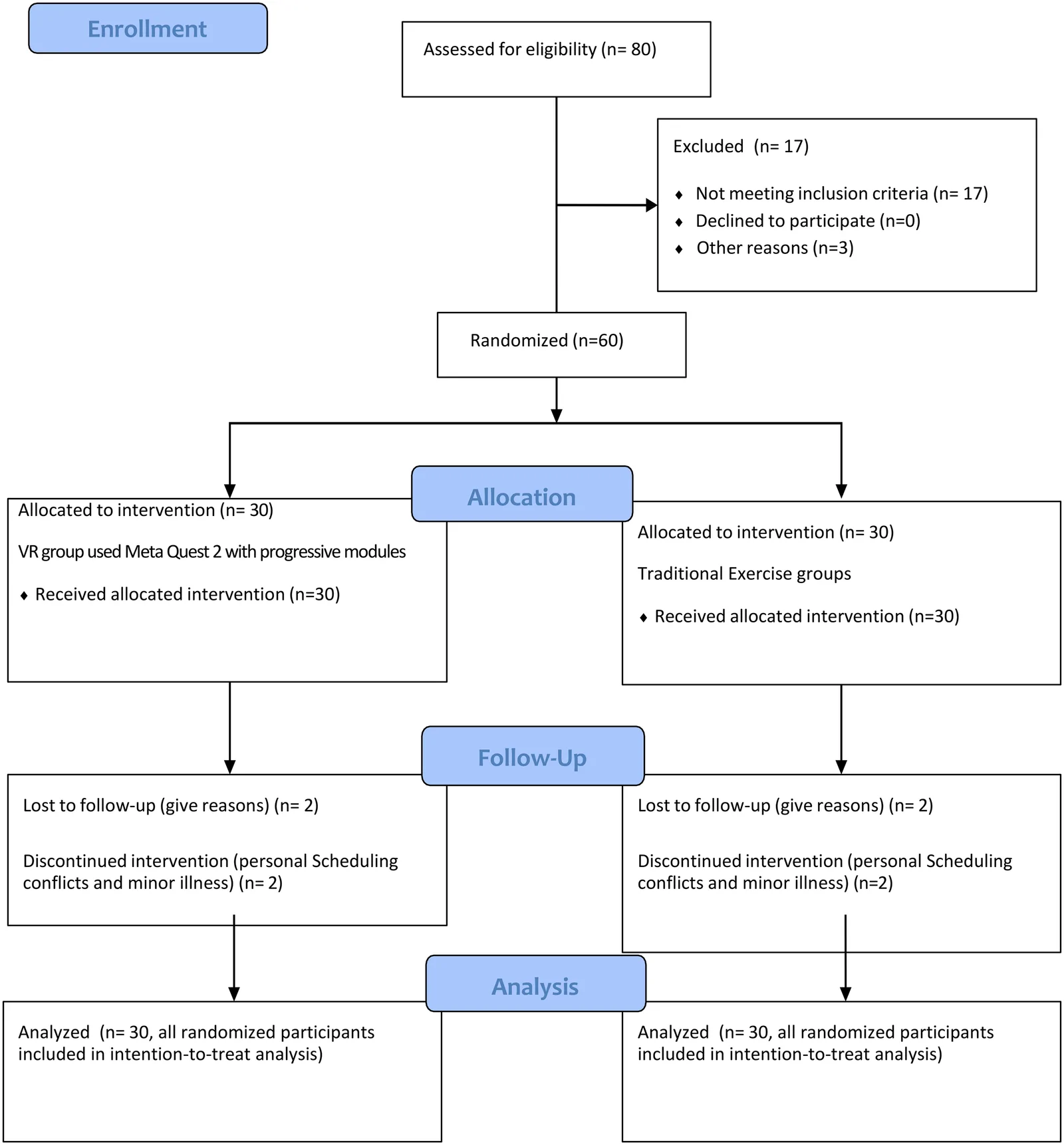
Consort flow chart.

**Blinding:** Outcome assessors and data analysts were blinded to group assignment. Participants and treating therapists were not blinded due to the nature of the interventions illustrated in Fig 1

### Sample size calculation

This study is a secondary analysis of a randomized controlled trial originally powered for the primary outcome of postpartum LPP, assessed using the Visual Analog Scale (VAS). Sample size was calculated for an independent groups t-test comparing change scores between the intervention and control groups. As no prior studies had evaluated VR-based exercise in postpartum women with LPP, proxy data were taken from a comparable randomized controlled trial evaluating core stability exercises in postpartum women. The reported mean and standard deviation of VAS scores (32.35 ± 12.51) were used to estimate the variability of change scores in the control group. Assuming a correlation of 0.73 between repeated measures, the standard deviation of the change score was estimated as 8.62.

A conservative assumption of no change in the intervention group (mean change = 0) was made, resulting in an expected between-group mean difference of 13.53. Using this difference and the pooled standard deviation of 12.19, the standardized effect size (Cohen’s d) was calculated as 1.11. With a two-sided alpha of 0.05 and 90% power, the minimum required sample size was 17 participants per group, calculated using the WHO sample size calculator for two independent means. To account for potential attrition and improve statistical robustness, the final sample size was increased to 30 participants per group (total n = 60) [11].

### Participants

A total of 60 postpartum women with clinically confirmed LPP, aged 20–40 years, were enrolled between 2 and 12 weeks after delivery. No participants outside this window were included. Eligibility screening was performed by a licensed physiotherapist, and participants were selected using simple random sampling. Both the primiparous and multiparous women were eligible.

### Inclusion criteria

Diagnosis required a positive result in at least three of six standardized clinical tests as follows:

Active straight leg raise (ASLR) – evaluates pelvic and lower limb stability by lifting one leg while supine [12]. The pelvic compression test, involving compression of the pelvis, was used to test pain that was localized to the sacroiliac joints [13]. Pelvic distraction test – applies outward pressure on the anterior pelvis to evaluate SI joint involvement [14]. Sacral thrust test – downward pressure on the sacrum to provoke SI joint pain [15].Posterior pelvic pain provocation – flexion of the hip to 90° with pressure along the femur to detect posterior pelvic pain [16].FABER Test – flexion, abduction, and external rotation of the hip to assess hip and sacroiliac joint dysfunction [17].

### Exclusion criteria

Women were excluded if they presented nausea, dizziness, or blurred vision; high-risk pregnancies; structural spinal disorders (scoliosis, kyphosis, lordosis); traumatic, inflammatory, or infectious conditions; diagnosed psychological disorders; history of spinal, pelvic, or femoral surgery or fractures; neoplastic conditions; or prior episodes of back pain.

### Intervention

**Fig 2:**
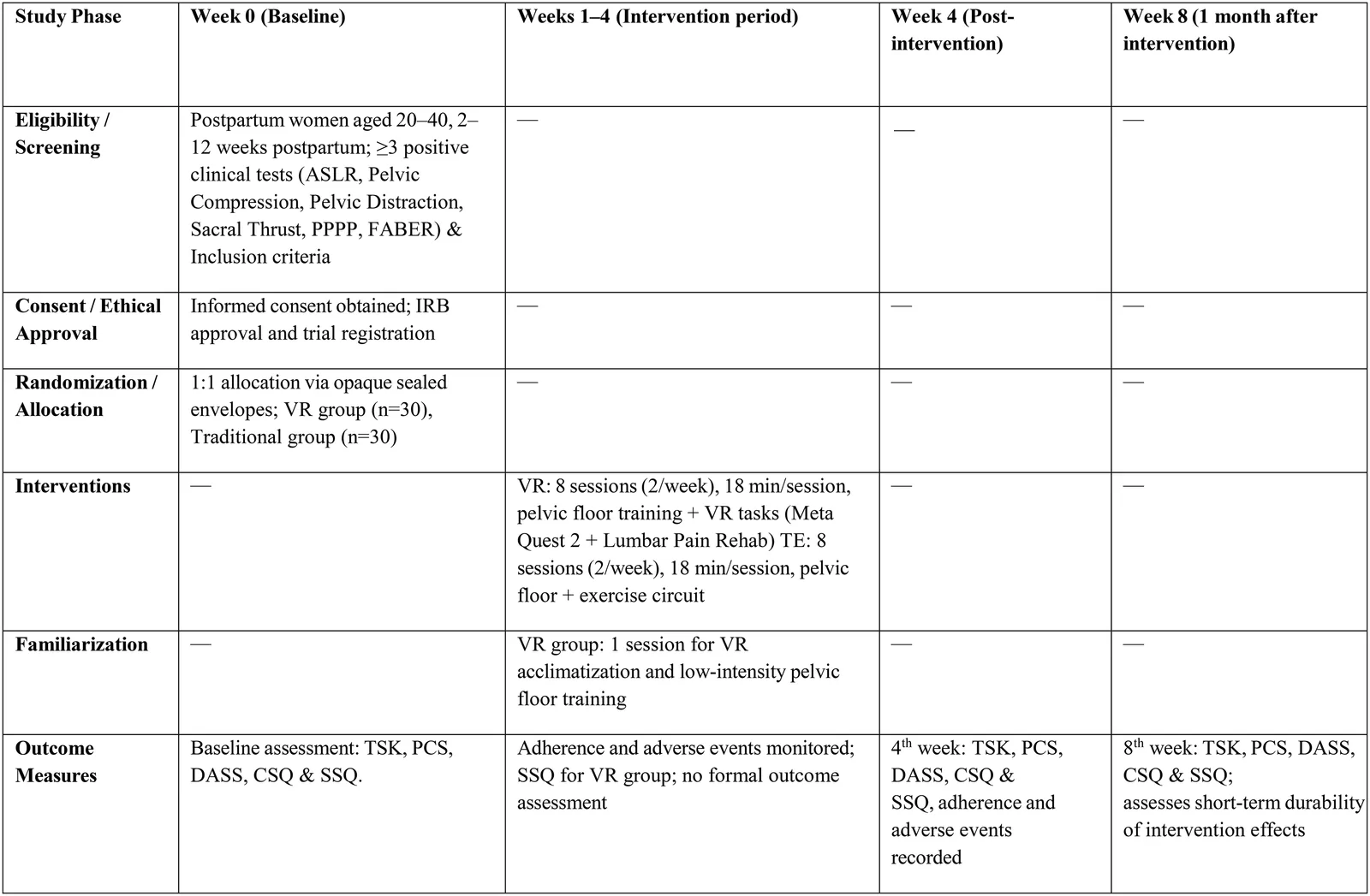
SPIRIT Schedule of Enrollment, Interventions, and Assessments.

Participants completed eight physiotherapy sessions over four weeks (two sessions per week). Session duration and intensity were progressively increased as detailed in the Procedure section. Adherence was monitored for each participant, and those who missed two or more sessions in a row were classified as dropouts. Reasons included personal scheduling conflicts and minor illness. Both interventions (VR and TE) aimed to reduce postpartum LPP.The study protocol adhered to Standard Protocol Items: Recommendations for Interventional Trials (SPIRIT) guidelines, with enrollment, allocation, intervention, and assessment steps structured accordingly illustrated in Fig 2.

### Procedure

#### Traditional exercise (TE) – control group.

An 18-minute training session was delivered, beginning with pelvic floor muscle training: 3 sets of 12 repetitions, with 3-second contractions and 3-second relaxations in a supine position. The second part included 2 sets of 5-minute training modules, with 1-minute rest between modules. The participants worked through a five-exercise circuit where each exercise lasted one minute to get as many repetitions as possible and then moved to the next Exercise. Two 1-kg dumbbells were required for these exercise protocols. The circuit comprised kegel pelvic floor exercises, deadlifts, wide-grip back rows, and lunges with trunk rotation, good-morning (hip hinge) exercises, and lateral side bends. These TE were aimed to improve pelvic floor strength and enhancing lumbopelvic function, reflecting evidence-based rehabilitation protocols for LPP [18].

#### VR as intervention group.

The participants in the intervention group were exposed to a VR exercise program with the help of Meta Quest 2 head-mounted displays. The VR intervention program was composed of eight guided sessions aimed at treating postpartum LPP by use of immersive interactive training. Pelvic muscle training with VR of 3 sets of 12 repetitions, three-second contraction relaxation cycles, and two 5-minute blocks of immersive VR-based activities with a 2-minute rest period, was included in each session, which entirely lasted 18 minutes.

The familiarization session was carried out before the intervention in order to acclimatize the participants to the VR environment. This involved modified pelvic floor training (3 sets of 8 repetitions with 3-second contraction/relaxation), a low-back-pain VR module (History Mode, Level 1) featuring 3 minutes of gameplay in an asymmetrical foot-standing position with slow-speed bending movements at one-third of the available range of motion (ROM), and 1-minute of reaction-speed training using two colored stimuli with 1-second obstacle intervals.

The intensity and complexity of the training were subsequently escalated in later sessions. Each session consists of standard pelvic floor training, followed by a low-back-pain module progressing from Level 2 to Level 9 in History Mode. These modules involved a gradual increase of movement speeds (slow, medium, fast), postures (e.g., asymmetrical pose), and movement patterns such as flexion, extension, and lateral inclination, rotation, and pelvic tilt. ROM grew at a slow pace of one-third to full range during the program. Every session was followed by 2 minutes of reaction speed training, with 1-second responses to two color visual stimuli and a moving obstacle

### Outcome measures

The main outcome of the parent randomized controlled trial was pain intensity measured with the help of the Visual Analog Scale (VAS); nevertheless, the given manuscript is based on kinesiophobia and corresponding patient-reported outcomes, which are examined as secondary and exploratory analyses based on the same cohort.

#### Primary outcome.

The primary outcome was kinesiophobia for the present study and was assessed using the Tampa Scale for Kinesiophobia (TSK) [19], a 17-item self-report instrument scored on a 4-point Likert scale, where higher scores indicate greater fear of movement or re-injury. Each item ranges from 1 to 4, interpreting strongly disagree to agree, making a total score from 17 to 68. Greater scores reflect increased kinesiophobia.

#### Secondary outcomes.

Pain catastrophizing was assessed using the Pain Catastrophizing Scale (PCS) [20], a 13-item self-report questionnaire scored on a 5-point Likert scale (0 = not at all to 4 = all the time). The PCS consists of three subdomains: rumination, magnification, and helplessness. Higher scores indicate increased catastrophic thinking about pain.

Emotional Distress, which has been defined as adverse emotional conditions, such as depression, anxiety, and stress, was assessed through the use of the Depression, Anxiety, and Stress Scale (DASS) [21]. This tool provides separate subscale scores, with established severity cut-offs: Normal: 0–9, Mild: 10–13, Moderate: 14–20, Severe: 21–27, Extremely Severe: ≥ 28

Pain coping strategies were measured using the Coping Strategies Questionnaire (CSQ) [22], which assesses three domains: cognitive, emotional, and behavioral responses to pain. Items are recorded on a 0–6 scale, with higher values shows more frequent use of each coping strategy.

Simulator-related symptoms were monitored using the Simulator Sickness Questionnaire (SSQ) [23], a validated tool assessing nausea, oculomotor strain, and disorientation in immersive VR environments. SSQ was included to ensure that VR exposure was well tolerated and did not adversely affect participant engagement.

### Statistical analysis

All statistical analyses were performed using IBM SPSS Statistics (version 26). Data distribution was assessed for normality using the Shapiro–Wilk test. Continuous variables that satisfied parametric assumptions were summarized as mean ± standard deviation (SD), while categorical variables were reported as frequencies and percentages.

To evaluate the effects of the interventions over time, a two-way mixed-design repeated-measures analysis of variance (ANOVA) was conducted for each continuous outcome, including Pain Catastrophizing Scale (PCS) subscales (Rumination, Magnification, and Helplessness), Coping Strategies, and Kinesiophobia. The model included group (VR vs TE) as the between-subject factor and time (baseline, 4^th^ week, and 8^th^ week) as the within-subject factor.

The primary effects of interest were the main effect of group, the main effect of time, and the group × time interaction effect, which reflects differential changes in outcomes across time between the two interventions. These effects were reported with corresponding p-values and partial eta squared (ηp²) as measures of effect size. Effect sizes were interpreted as small (~0.01), medium (~0.06), and large (~0.14), in accordance with established conventions.

Assumptions of sphericity were evaluated using Mauchly’s test of sphericity. When the assumption of sphericity was violated, the Greenhouse–Geisser correction was applied to adjust degrees of freedom. Where significant main or interaction effects were observed, post hoc pairwise comparisons were performed using estimated marginal means with Bonferroni adjustment to control for multiple comparisons.

For categorical outcomes, including levels of depression, anxiety, and stress (DASS categories), between-group differences at each time point were analysed using the chi-square test.

The Simulator Sickness Questionnaire (SSQ), which was administered only to participants in the VR group, was analysed separately using within-group repeated-measures ANOVA to assess changes over time. Between-group and interaction analyses were not applicable for this outcome due to the absence of a control group.

Missing data from four participants who discontinued before the 8^th^ week follow-up were handled using the Last Observation Carried Forward (LOCF) method. While this approach is commonly used in clinical trials, it has known limitations, including potential bias in variance estimation and treatment effect inflation; therefore, results were interpreted with appropriate caution.

A two-tailed p-value < 0.05 was considered statistically significant for all analyses.

## Results

All randomized participants (n = 60) were included in the primary analyses according to the intention-to-treat (ITT) principle. Four participants discontinued before the 8^th^ week, and missing data were handled using the LOCF method. A peer-control sensitivity analysis, excluding participants with ≥2 missed sessions, yielded similar results, supporting the robustness of the findings

Table 1 presents baseline demographic and clinical characteristics, which were comparable between the VR (Group A) and TE (Group B) groups. Age distribution was identical, with 36.7% of participants aged ≤28 years and 63.3% aged >28 years in both groups (p > 0.999). Body mass index was evenly distributed (p = 0.935), and baseline pelvic pain (56.7 vs. 50.0; p = 0.605) and pre-delivery urinary incontinence (6.7% in both groups; p = 0.999) showed no significant differences. A significant baseline imbalance was observed in mode of delivery, with more caesarean sections in the VR group (90.0%) compared to the TE group (66.7%; p = 0.028). All subsequent analyses were adjusted for mode of delivery to account for this potential confounder. This study utilized the same cohort as previously reported in an accepted manuscript.

**Table 1:** Comparison of baseline characteristics between the Experimental and Control Group.

		Group			
**Baseline Information**	**Categories**	**Group A**	**Group B**		**p-value**
Age group	≤28 years	11(36.7%)	11(36.7%)	22(36.7%)	>0.999
	> 28 years	19(63.3%)	19(63.3%)	38(63.3%)	
Body Mass Index	Normal	11(36.7%)	12(40.0%)	23(38.3%)	0.935
	Overweight	13(43.3%)	13(43.3%)	26(43.3%)	
	Obese	6(20.0%)	5(16.7%)	11(18.3%)	
Pelvic pain		17(56.7%)	15(50.0%)	32(53.3%)	0.605
Mode of delivery	SVD	3(10.0%)	10(33.3%)	13(21.7%)	0.028
	C-Section	27(90.0%)	20(66.7%)	47(78.3%)	
Incontinence prior to delivery		2(6.7%)	2(6.7%)	4(6.7%)	>0.999

Group A, VR group (Experimental), Group B TE (Control) group.

Table 2 summarizes outcome measures across baseline, 4^th^ and 8^th^ week assessments. Both groups showed significant within-group improvements in depression, anxiety, stress and simulator-related symptoms (p < 0.001).

**Table 2:** Between- Group Analysis of Pre- and Post-Intervention Outcomes: Assessing the Psychological and Emotional Benefits of Virtual Reality Versus Traditional Exercise Programs in Postpartum Women.

Characteristics			Experiment VR Group	Control TE Group	p-value	Effect Size (r)
**DEPRESSION**	Baseline	Mild	3(10%)	4(20%)	0.830^‡^	0.072
		Moderate	16(53.3%)	17(56.6%)		
		severe	11(36.6%)	9(30%)		
	4^th^ Week	Normal	2(6.67%)	3(10%)	0.007^‡^	0.150
		Mild	19(63.3%)	7(56.6%)		
		Moderate	9(30%)	20(66.6%)		
	8^th^ Week	Normal	10(33.3%)	10(33.3%)	0.033^‡^	0.30
		Mild	14(46.6%)	5(16.6%)		
		Moderate	6(20%)	13(43%)		
**ANXIETY**	Baseline	Mild	3(10%)	6(20%)	0.499^‡^	0.068
		Moderate	13(43%)	10(33.3%)		
		severe	14(46.6%)	14(46.6%)		
	4^th^ Week	Mild	19(63%)	10(33.3%)	0.038^‡^	0.18
		Moderate	11(36.6%)	20(66.6%)		
	8^th^ Week	Normal	24(80%)	19(63%)	0.20^‡^	0.23
**STRESS**	Baseline	Normal	5 (16.7%)	5 (16.7%)	1.000^‡^	0.000
		Mild	12 (40.0%)	12 (40.0%)		
		Moderate	13 (43.3%)	13 (43.3%)		
	4^th^ Week	Normal	19 (63.3%)	17 (56.7%)	0.856^‡^	0.072
		Mild	9 (30.0%)	11 (36.7%)		
		Moderate	2 (6.7%)	2 (6.7%)		
	8^th^ Week	Normal	24 (80.0%)	23 (76.7%)	0.508^‡^	0.150
		Mild	5 (16.7%)	7 (23.3%)		
		Moderate	1 (3.3%)	0 (0.0%)		
**Simulator Sickness Questionnaire (SSQ)**	Baseline		7.79 ± 1.80	not aplicable for control group	not applicable	0.658
	4^th^ Week		4.34 ± 2.11			
	8^th^ Week		3.19 ± 2.28			
	
	WITHIN P-VALUE		0.001¥			

Note: - Chi-square test (‡), A *p*-value <0.05 was considered significant.

Baseline depression scores did not differ significantly between groups (36.6% of VR participants scored as severe versus 30% in control; p = 0.830). By the 4^th^ week, 6.67% of VR participants improved to the normal range vs 10% in control (p = 0.007, r = 0.15). By the 8^th^ week, 33.3% of participants in both groups were classified as normal, while the VR group had a higher proportion in the mild category (46.6% vs 16.6%; p = 0.033, r = 0.30).

Baseline anxiety levels were not significantly different (p = 0.499, r = 0.068). By the 4^th^ week, the VR group showed a higher proportion in the mild anxiety category (63% vs 33.3%; p = 0.038, r = 0.18), and by the 8^th^ week, 80% of VR participants fell within the normal category compared with 63% in TE (p = 0.20, r = 0.23).

Simulator-related symptoms in the VR group were minimal and decreased over time, indicating good tolerance to repeated VR exposure. No adverse effects that interfered with participation were reported, supporting the feasibility and acceptability of VR-based rehabilitation in this postpartum population.

Adjustment for mode of delivery did not materially alter the magnitude or direction of intervention effects, and no significant interaction between intervention group and mode of delivery was observed for any outcome.

Table 3 presents the longitudinal effects of Virtual Reality (VR) and Traditional Exercise (TE) on psychological outcomes among postpartum women. Mixed-design repeated-measures ANOVA demonstrated significant time effects for all psychological outcomes. Large effect sizes were observed for time across all outcomes (ηp² = 0.638–0.955.

**Table 3:** Longitudinal Effects of VR vs Traditional Exercise on Psychological Outcomes in Postpartum Women.

Outcome	VR Baseline (Mean ± SD)	VR Week 4	VR Week 8	TE Baseline	TE Week 4	TE Week 8	Group p	ηp² (Group)	Time p	ηp² (Time)	Group × Time p	ηp² (Interaction)	Post hoc (Bonferroni)
PCS-R	11.20 ± 1.10	8.30 ± 1.25	7.10 ± 1.05	11.10 ± 1.15	9.20 ± 1.30	9.40 ± 1.20	0.113	0.043	<0.001	0.638	<0.001	0.176	Baseline > Week 4 > Week 8
PCS-M	9.10 ± 0.90	6.10 ± 0.80	5.20 ± 0.70	9.00 ± 1.00	7.20 ± 0.90	6.30 ± 0.85	<0.001	0.200	<0.001	0.955	<0.001	0.576	All time points differ
PCS-H	21.30 ± 1.50	10.40 ± 1.60	8.20 ± 1.40	21.10 ± 1.40	12.10 ± 1.70	10.30 ± 1.60	<0.001	0.215	<0.001	0.952	<0.001	0.184	Baseline > Week 4 > Week 8
Coping Strategies	2.80 ± 0.25	3.05 ± 0.28	3.35 ± 0.30	3.05 ± 0.27	3.22 ± 0.29	3.35 ± 0.31	0.001	0.045	<0.001	0.810	0.020	0.329	Baseline vs Week 8
Kinesiophobia	55.5 ± 6.0	35.5 ± 5.5	28.5 ± 4.8	56.5 ± 6.2	50.0 ± 5.8	46.5 ± 5.0	<0.001	0.248	<0.001	0.750	<0.001	—	All time comparisons significant

**Abbreviations:** PCS-R = Pain Catastrophizing Scale–Rumination; PCS-M = Pain Catastrophizing Scale–Magnification; PCS-H = Pain Catastrophizing Scale–Helplessness; TE = Traditional Exercise; VR = Virtual Reality; ηp² = partial eta squared. Continuous variables are presented as mean and standard deviation. Group, time, and interaction effects were analyzed two-way mixed-design repeated-measures analysis of variance (ANOVA). Post hoc comparisons were adjusted using Bonferroni correction. A p-value < 0.05 was considered statistically significant. LOCF was used for handling missing data.

For PCS-R (Rumination), a significant effect of time (p < 0.001, ηp² = 0.638) and a significant group × time interaction (p < 0.001, ηp² = 0.176) were observed, indicating that reductions in rumination differed between groups over time. However, the overall between-group effect was not significant (p = 0.113, ηp² = 0.043).

For PCS-M (Magnification), significant group (p < 0.001, ηp² = 0.200), time (p < 0.001, ηp² = 0.955), and group × time interaction effects (p < 0.001, ηp² = 0.576) were found.

Similarly, PCS-H (Helplessness) demonstrated significant group (p < 0.001, ηp² = 0.215), time (p < 0.001, ηp² = 0.952), and group × time interaction effects (p < 0.001, ηp² = 0.184). The large time effect suggests marked reductions in helplessness throughout the intervention, while the interaction effect indicates improvement in the VR group.

For Coping Strategies, significant effects were observed for group (p = 0.001, ηp² = 0.045), time (p < 0.001, ηp² = 0.810), and the group × time interaction (p = 0.020, ηp² = 0.329). These results suggest that coping abilities improved over time, with the VR intervention producing more gains than traditional exercise.

Regarding Kinesiophobia, significant reductions were observed across time (p < 0.001, ηp² = 0.750), accompanied by a significant group effect (p < 0.001, ηp² = 0.248). Participants in the VR group demonstrated reductions in fear of movement compared with the traditional exercise group. The noteworthy interaction effect further indicates that the pattern of improvement over time differed between interventions, favouring VR

## Discussion

The current study compared VR-based and TE programs in postpartum women with LPP. The two interventions demonstrated great short-term effects on psychological outcomes, such as kinesiophobia, pain-related cognitive patterns, emotional distress (depression and anxiety), and coping modes. Nevertheless, participants in the VR group showed a favorable trend toward greater improvement in fear of movement and maladaptive pain-related cognitive responses compared with the TE group. Pain catastrophizing reductions in the VR group were considered significant, representing changes in cognitive domains, including helplessness, magnification, and rumination. Both groups also exhibited improvements in emotional well-being, with VR participants demonstrating a more desirable change in the direction of reduced levels of anxiety and depressive symptoms at the 4^th^ and 8^th^ weeks. Both interventions had increased coping responses over time, with subjects in the VR group showing faster gains, which eventually evened out and matched those of the TE group. Moreover, the VR intervention was tolerable, and the participants adapted well to the virtual environment. Overall, these findings suggest that while both rehabilitation approaches contribute to early psychological improvements in postpartum LPP, immersive VR may provide additional benefits in reducing fear of movement and maladaptive pain-related cognitive patterns.

Both TEs and VR-based interventions showed significant differences in fear of movement by postpartum women with LPP. Nevertheless, there was a higher reduction in kinesiophobia in the VR intervention than in TE, implying that immersive rehabilitation might be extremely helpful in the treatment of fear associated with movement. The observed improvements exceeded established thresholds for clinically meaningful change [24], indicating that these reductions were relevant for functional recovery. These findings align with previous research demonstrating that combining VR with physical therapy significantly reduced fear of movement (TSK scores) compared with physical therapy alone in patients with chronic pain, indicating the potential of VR to alleviate movement-related fear [25]. Additionally, evidence from meta-analytic studies indicates that both immersive and non-immersive VR-assisted active training are effective in reducing chronic neck and back pain, supporting its potential to alleviate movement-related fear and enhance functional recovery in musculoskeletal pain populations [26].

TE-based rehabilitation, such as core stabilization and strengthening programs, has also been proven to be effective at reducing fear of movement and positively influencing functional performance in musculoskeletal pain patients. Traditional rehabilitation research indicates that exercise interventions based on stabilization can have a beneficial impact on kinesiophobia in individuals with chronic low back pain [27]. In the same way, core stabilization and strengthening training have been linked to the decrease in fear of movement combined with enhancement of balance and pain outcomes, emphasizing the value of organized physical rehabilitation in managing movement-related fear [28]. When considered together with the effects observed in the VR intervention, these findings suggest that both immersive and exercise-based rehabilitation strategies may contribute to improving movement confidence and functional recovery. Although direct evidence in postpartum women with LPP remains limited, the present findings support the potential applicability of these approaches within postpartum rehabilitation.

Postpartum women with LPP showed meaningful reductions in maladaptive pain cognitions following both VR-based and TE interventions. Improvements were particularly notable in feelings of helplessness, with the VR intervention providing additional benefits compared with TE [29]. The VR intervention provided additional benefits in alleviating maladaptive cognitive responses to pain, particularly in reducing feelings of helplessness. These findings suggest that immersive VR may be especially effective in targeting negative pain-related thoughts, with the potential for further improvements in other cognitive domains over time.

The results showed that the VR intervention may provide enhanced psychological benefits compared to TE, especially in terms of reducing pain catastrophizing and enhancing adaptive pain-related cognition. Immersive VR likely modulates pain by facilitating multisensory integration and redirecting attention toward immersive external cues, thereby reducing the activation of affective pain-processing areas such as the anterior cingulate cortex and insula [30]. In this context, VR may act as a cognitive distractor capable of interrupting repetitive negative thoughts, promoting a stronger sense of control, and potentially reducing feelings of helplessness. These mechanisms may help explain the significant reductions in catastrophizing observed in the VR group. Recent systematic reviews support that the efficacy of virtual reality–based interventions can effectively reduce pain catastrophizing, particularly when high-immersion formats and psychocognitive components are employed, supporting the potential of VR to address maladaptive pain-related cognitions in chronic pain populations. Moreover, randomized evidence indicates that combining VR with physical therapy produces greater reductions in PCS scores than exercise alone in patients undergoing total knee arthroplasty, highlighting the additive therapeutic value of immersive, interactive interventions [31].

Improvements observed in the TE group align with previous research by Sullivan [32], who examined adult patients with chronic low back pain receiving standard physical therapy and reported significant reductions in pain-catastrophizing scores, suggesting that conventional physiotherapy can positively influence maladaptive pain cognitions in this population. Similarly, a pilot study investigating structured core-stability and exercise programs in adults with nonspecific chronic low back pain reported moderate reductions in catastrophizing and improvements in pain-related disability, highlighting the broader benefits of targeted exercise interventions in chronic pain management [33]. Moreover, emerging evidence suggests that combining physiotherapy with VR may provide complementary benefits beyond exercise alone. For example, Sakuma demonstrated that a physiotherapy program augmented with VR produced larger reductions in pain‑related fear of movement and was associated with greater improvements in pain catastrophizing compared with conventional physiotherapy in patients with chronic pain [25], indicating potential additive psychological benefits when immersive environments are integrated into rehabilitation. While direct evidence in postpartum women remains limited, these studies collectively support the potential value of combining immersive VR with structured rehabilitation to enhance psychological outcomes in musculoskeletal pain populations. Further research is warranted to assess whether these benefits are maintained over longer-term follow-up in postpartum populations.

Postpartum women in the VR group demonstrated significant improvements in depressive and anxiety symptoms as measured by the DASS, while baseline severity distributions were comparable between groups. By the 4^th^ week, a greater proportion of participants in the VR group experienced improvements in depression and anxiety compared with the control group, indicating that immersive VR may provide additional benefits in alleviating psychological distress. Over time, both groups continued to improve, with the VR group showing a stronger early response and a larger shift toward normative levels of anxiety and mood, suggesting that VR may accelerate psychological recovery in postpartum women. These findings indicate that immersive VR provided additional short-term benefit in alleviating psychological distress, particularly in reducing depressive and anxiety symptoms, with potential for further improvements over longer follow-up.

Immersive VR may alleviate depressive and anxiety symptoms by facilitating relaxation through calming environments, thereby reducing psychological distress and supporting emotional regulation. This interpretation is consistent with evidence showing that immersion in simulated natural environments can attenuate stress and negative affect [34]. Similarly, VR-enhanced mindfulness and yoga interventions have demonstrated benefits for maternal mental health, highlighting the potential of immersive VR as a supportive tool for postpartum women [10]. The improvements observed in the TE group are consistent with systematic review evidence indicating that postpartum exercise reduces depressive and anxiety symptoms, underscoring physical activity as a key component of psychological recovery in new mothers [35]. Together, these findings suggest that immersive VR produced moderate short-term improvements in depressive and anxiety symptoms, particularly by shifting participants toward lower severity categories. However, although the one-month follow-up results are encouraging, longer-term follow-up is required to determine whether these effects are sustained and to assess the potential for VR-based interventions to promote lasting improvements in maternal mental health.

The study examined the effects of immersive VR and TE on pain-related coping strategies. Participants in the VR group demonstrated consistent improvements in coping over the course of the intervention, eventually reaching levels comparable to those in the exercise group. These findings suggest that immersive VR may enhance adaptive coping in postpartum women with LPP, particularly by accelerating early gains, although the relative advantage diminished as both groups achieved similar outcomes by the 4^th^ and 8^th^ week.

Maladaptive coping is often characterized by passive and emotion-focused responses to stress, which can limit effective adjustment during the postpartum period [36]. Immersive virtual reality (VR) may promote more adaptive coping by engaging attentional control and emotion-regulation processes, thereby enhancing cognitive reappraisal and encouraging active stress-management strategies. This interpretation is supported by evidence demonstrating that immersion in simulated natural environments reduces stress and emotional arousal—mechanisms closely associated with improved coping capacity [34]. In addition, VR-based interventions have been shown to reduce pain catastrophizing and enhance self-efficacy, reflecting improvements in cognitive coping strategies through immersive and engaging therapeutic experiences [37].

In the present study, the VR group demonstrated significant within-group improvements in coping strategies over time, progressing from lower baseline scores to levels comparable with the control group at both the 4^th^ and 8^th^ week. These findings suggest that VR may yield moderate short-term benefits in enhancing coping strategies, particularly by accelerating early gains in cognitive coping. Improvements observed in the TE group are consistent with evidence showing that physical activity enhances psychological resilience and emotional regulation, thereby supporting adaptive coping in postpartum women [35]. While both interventions appear beneficial, future studies with extended follow-up are required to assess the durability of VR-related improvements in coping strategies over time.

The Simulator Sickness Questionnaire (SSQ) was used to monitor the adverse effects of VR exposure in the experimental group. Participants tolerated the intervention well and reported discomfort decreased over repeated sessions. This pattern is consistent with published evidence showing that VR-related symptoms generally decline as users adapt to immersive environments [38,39]. Overall, these results indicate a favorable tolerability profile, supporting the feasibility and safe implementation of VR-based rehabilitation in postpartum populations, and suggesting that VR can be integrated into clinical protocols with minimal risk of simulator-induced adverse effects.

Although the present study did not evaluate the cost-effectiveness of immersive VR, its implementation in postpartum rehabilitation requires consideration of both the initial investment and potential long-term value. The use of VR involves costs related to head-mounted displays, software, equipment maintenance, and staff training, which may limit accessibility in some healthcare settings, particularly in low- and middle-income countries [40]. However, as VR technology becomes increasingly available and affordable, these costs may be offset by improved patient engagement, adherence to rehabilitation programs, and the potential for more efficient delivery of exercise interventions [41]. Given that postpartum women often encounter barriers to accessing rehabilitation services, immersive VR may offer a practical adjunct to conventional physiotherapy. Future studies should incorporate formal economic evaluations to determine the cost-effectiveness of VR-based rehabilitation and its feasibility for wider implementation in maternal healthcare.

In summary, both immersive VR and TE contributed to improvements in physical and psychological outcomes among postpartum women with LPP. Immersive VR appeared to facilitate early enhancements in fear of movement, maladaptive pain cognitions, and coping strategies, potentially accelerating cognitive and affective adaptation during rehabilitation. TE also supported improvements in psychological well-being and functional recovery, highlighting the value of structured physical activity in postpartum care. Together, these findings suggest that integrating immersive VR into rehabilitation programs may provide additional engagement and interactive benefits, complementing conventional exercise approaches. Future research with larger sample sizes and extended follow-up is warranted to determine the durability of these effects and to clarify the long-term clinical relevance of VR-based interventions in postpartum populations.

### Limitation

This study has several limitations, the follow-up period was limited to 8 weeks, which restricts assessment of the long-term sustainability and clinical relevance of the observed effects. Therefore, the durability of improvements in psychological outcomes and pain-related cognitions beyond the intervention period remains uncertain. All outcomes were based on self-reported questionnaires, which may be subject to response and reporting bias. Although validated instruments were used, they do not capture underlying physiological, neurocognitive, or behavioral mechanisms associated with the observed changes. Missing data were handled using the Last Observation Carried Forward (LOCF) method. While this approach supports intention-to-treat analysis, it may introduce bias by assuming outcome stability after dropout and potentially underestimating variability or treatment effects.

In addition, multiple outcomes and repeated statistical tests were performed across several time points. Although appropriate corrections (e.g., Bonferroni adjustment) were applied for post hoc analyses, the risk of type I error inflation cannot be fully excluded. Therefore, p-values should be interpreted with caution, particularly for secondary and exploratory outcomes.

Finally, the sample was relatively small and drawn from a single clinical setting, which may limit generalisability to broader postpartum populations. Furthermore, this study did not include a formal economic or cost-effectiveness evaluation of the immersive VR intervention; therefore, future studies should assess its economic feasibility alongside clinical effectiveness.

### Practical implications

Immersive VR and structured exercise provide complementary benefits in postpartum rehabilitation, with VR supporting improvements in fear of movement, maladaptive pain cognitions, and coping, and exercise reinforcing physical conditioning and lumbopelvic stability. In order to produce meaningful and lasting change in psychological and functional outcomes, interventions must be deliberately designed to focus on both physical and mental health. A proper choice of VR content, exercise type, and timeline of the sessions can help to achieve optimal engagement, decrease discomfort, and enhance functional and psychological benefits. These considerations can guide clinicians in integrating VR alongside conventional exercise to enhance the postpartum rehabilitation program.

### Future directions

Future research should focus on developing standardized VR protocols and evaluating their long-term effects on pain, psychological outcomes, and coping strategies. Generalizability should be confirmed by conducting studies that involve larger and more diverse groups of postpartum. Moreover, further studies on cost-effectiveness and remote or hybrid delivery models, as well as customized VR functions, may contribute to the further expansion of VR as a standard element of postpartum rehabilitation.

## Conclusion

Immersive VR and TE were effective for postpartum rehabilitation, producing improvements in pain-related fear, maladaptive cognitions, psychological symptoms, and coping strategies. VR demonstrated favourable short-term effects by accelerating early improvements in fear of movement, maladaptive pain cognitions, and coping strategies. Such findings place VR as a helpful addition to TE and increase participation and initial cognitive and psychological benefits. Such benefits need to be measured by long-term follow-up to define their sustainability and optimize VR protocols that can be included in postpartum care.

## Supporting information

S1 Checklist Inclusivity in Global Research. (DOCX)

S1 Table Two-way ANOVA examining effects of intervention group and mode of delivery on VAS at 8^th^ week Assessment. (ODT)

S1 Data Participant-level outcome measurements at baseline, 4th-week, and 8th-week assessments. (XLSX)
