# Supplementary material for: Psychological and emotional benefits of virtual reality-based exercise for postpartum women with lumbopelvic pain: A randomized controlled trial
Source: PLOS Glob Public Health. 2026 Sep 2;6(9):e0006354. doi: 10.1371/journal.pgph.0006354 (PMC13537561; doi:10.1371/journal.pgph.0006354)
Supplement: Supplementary file 2 [file pgph.0006354.s002.odt]

Two-way ANOVA examining effects of intervention group and mode of delivery on VAS at 8th week Assessment.”

Source
F
df
p
Partial η²
Group
26.342
1,56
<.001
0.320
Mode of Delivery
0.006
1,56
.938
0.000
Group × Mode of Delivery
2.939
1,56
.092
0.050

Note: F = F-statistic; Sig. = p-value; η²p = partial eta squared (effect size). p < 0.05 indicates statistical significance.
